# Cholestasis-induced phenotypic transformation of neutrophils contributes to immune escape of colorectal cancer liver metastasis

**DOI:** 10.1186/s12929-024-01052-3

**Published:** 2024-06-29

**Authors:** Li Sun, Nanyan Yang, Zhihong Liu, Xiandong Ye, Mengting Cheng, Lingjun Deng, Junhao Zhang, Jingjing Wu, Min Shi, Wangjun Liao

**Affiliations:** 1 Department of Oncology, Nanfang Hospital, Southern Medical University, Guangzhou, 510515 Guangdong China; 2 Foshan Key Laboratory of Translational Medicine in Oncology, Cancer Center, the Sixth Affiliated Hospital, South China University of Technology, Foshan, Guangdong, China; 3 Department of Oncology, Air Force Medical Center of PLA, Air Force Medical University, Beijing, China; 4 Department of Thoracic Medical Oncology, Cancer Hospital of the University of Chinese Academy of Sciences, Zhejiang Cancer Hospital, Hangzhou, Zhejiang China

**Keywords:** Bile acids, Cholestasis, Colorectal cancer liver metastasis, Neutrophil, Tumor microenvironment

## Abstract

**Background:**

Cholestasis is a common yet severe complication that occurs during the advancement of liver metastasis. However, how cholestasis impacts the development, treatment, and tumor microenvironment (TME) of liver metastasis remains to be elucidated.

**Methods:**

Extrahepatic and intrahepatic cholestatic mouse models with liver metastasis were established to detect the differential expression levels of genes, infiltration of immune cells and change in bile acid-associated metabolites by using RNA-Sequencing, flowcytometry, and liquid chromatography and mass spectrometry. Western blot was applied to neutrophils under the stimulation of primary bile acids (BAs) in vitro to study the mechanism of phenotypic alteration. In vitro coculture of BA-treated neutrophils with CD8^+^ T cells were performed to study the immune-suppressive effect of phenotypic-altered neutrophils. Clinical samples collected from colorectal cancer patients with liver metastasis and cholestasis were applied to RNA-Seq.

**Results:**

Compared to non-cholestatic mice, the progression of liver metastasis of cholestatic mice was significantly accelerated, which was associated with increased neutrophil infiltration and T-cell exclusion. Both neutrophils and T cells expressed higher immunosuppressive markers in the cholestatic mouse model, further indicating that an immunosuppressive tumor microenvironment was induced during cholestasis. Although neutrophils deletion via anti-Ly6G antibody partially hindered liver metastasis progression, it reduced the overall survival of mice. Tauro-β-muricholic acid (Tβ-MCA) and Glycocholic acid (GCA), the two most abundant cholestasis-associated primary BAs, remarkably promoted the expression of Arg1 and iNOS on neutrophils via p38 MAPK signaling pathway. In addition, BAs-pretreated neutrophils significantly suppressed the activation and cytotoxic effects of CD8^+^ T cells, indicating that the immunosuppressive phenotype of neutrophils was directly induced by BAs. Importantly, targeting BA anabolism with Obeticholic acid (OCA) under cholestasis effectively suppressed liver metastasis progression, enhanced the efficacy of immune checkpoint blockade, and prolonged survival of mice.

**Conclusions:**

Our study reveals the TME of cholestasis-associated liver metastasis and proposes a new strategy for such patients by targeting bile acid anabolism.

**Graphical Abstract:**

Schematic model depicting the proposed mechanism of cholestasis-mediated progression of colorectal liver metastasis.

As cholestasis progresses, excessive primary bile acids that accumulate in the liver intoxicates hepatocytes, which lead to exacerbated release of chemokines, particularly CXCL2 and CXCL5. Neutrophils are then accumulated by CXCL2 and CXCL5 and undergo an immunosuppressive-phenotypic alteration induced by direct stimulation of BAs via activating the p38 MAPK signaling pathway, which eventually led to the dysfunction of T cells and progression of LM. Targeting bile acid anabolism can effectively restore the immune-activated microenvironment and prevent the progression of LM.

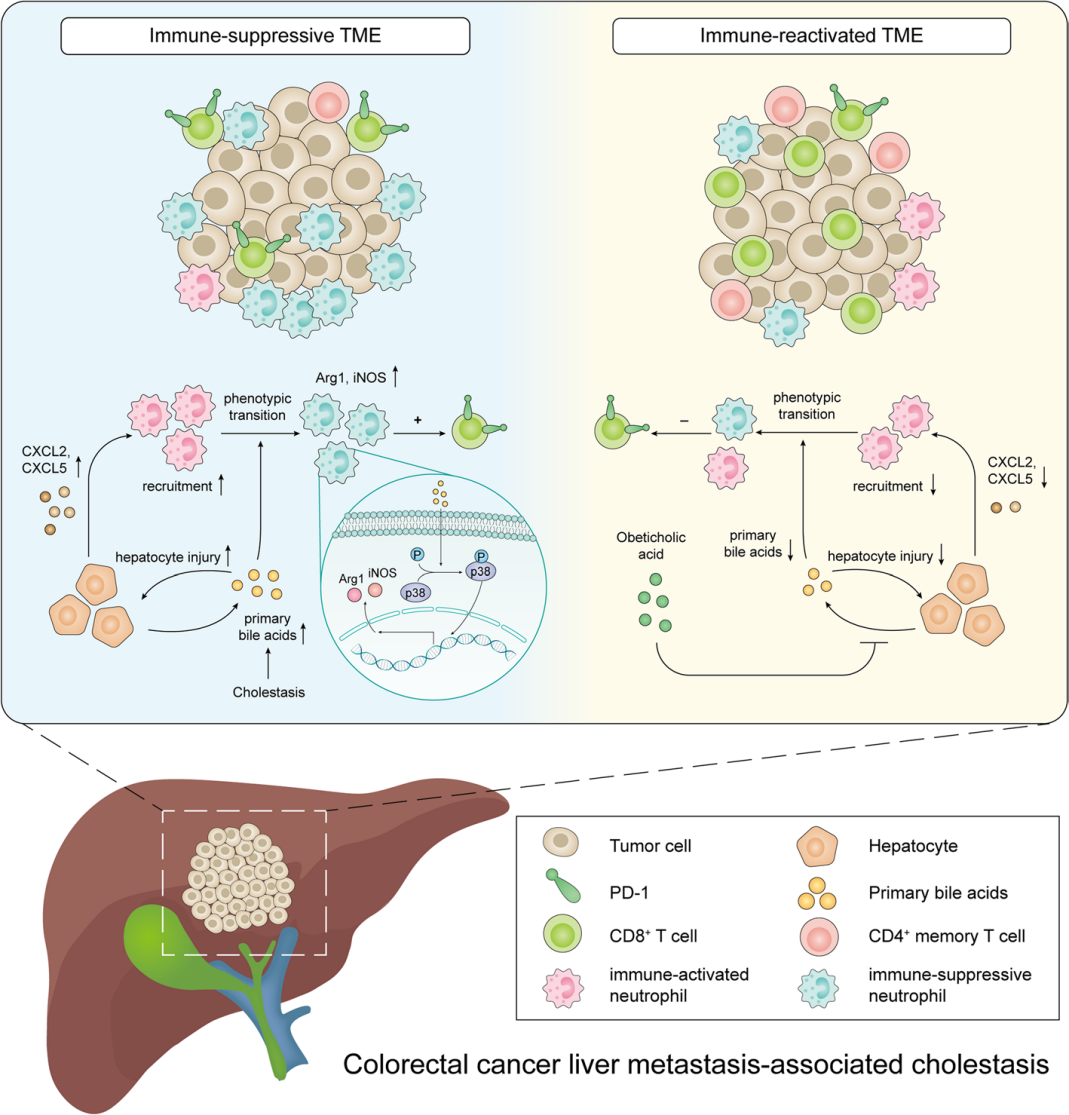

**Supplementary Information:**

The online version contains supplementary material available at 10.1186/s12929-024-01052-3.

## Background

Liver metastasis (LM) has become a major cause of death in colorectal cancer (CRC), stomach cancer, lung cancer, etc. Approximately 80–90% of patients with metastatic colorectal cancer are present with nonresectable LM [1]. Cholestasis, biliary obstruction or obstructive jaundice secondary to colorectal cancer liver metastasis (CRLM), ceased chemotherapy or other treatments for patients which further lead to poor prognosis. Median survival after the onset of jaundice is only around one month even when supported with care [2]. Unfortunately, there is a lack of efficient strategies to improve the clinical outcomes of patients, especially those who cannot undergo biliary drainage. Even the patients were to undergo functional biliary drainage, the median survival is less than four months [3]. Consequently, there is an urgent need to explore novel effective therapeutic strategies to improve the long-term survival of these patients.

The tumor microenvironment (TME) is constantly interacting with tumor cells, which significantly effects tumor development and tumor response to immune checkpoint blockade (ICB) or other interventions [4–7]. The TME is associated with various non-cancerous cells as well as tissue mechanical and chemical properties, which include various metabolites. The decompensation of bile acid metabolism is the core pathological feature of cholestasis, manifesting increased total bile acids (TBA) levels, impaired bile acid (BA) secretion, and accumulation of primary and secondary BAs in the liver [8]. Owing to the lipophilic and detergent properties of BAs, their excessive accumulation in the liver can cause liver injury and inflammation by disrupting the plasma membrane and activate a series of inflammatory signaling pathways, DNA damage, which eventually leads to hepatocarcinoma and CRC [8]. BAs and metabolites can influence various immune cells. For instance, the derivatives of lithocholic acid control the differentiation of T helper cells and regulatory T cells [9] and the activation of NLRP3 of bone-marrow derived macrophages [10]. The accumulation and function of natural killer T cell (NKT) cells is correlated with secondary bile acids [11]. Furthermore, in response to cholestatic liver injury, mast cells infiltrate the liver and activate hepatic stellate cells (HSCs) to increase the expression of α-smooth muscle actin (α-SMA), fibronectin-1, and collagen [12]. In primary biliary cirrhosis, myeloid-derived suppressive cells (MDSCs) are associated with liver fibrosis and dysfunction of cytotoxic T cells [13]. During bile duct obstruction, neutrophils can recruit and activate T helper cell that expresses IL-17α (Th17) that contributes to local inflammation [14]. Nonetheless, it is unknown about the change of bile acid spectrum in colorectal liver metastasis during cholestasis and how it in turn influences the TME.

In order to investigate the potential impact of cholestasis and dysregulation of bile acid metabolism on CRLM, we have established the extrahepatic and intrahepatic cholestatic liver metastatic mouse models and analyzed clinical data. Moreover, the role and mechanisms of neutrophils in establishing the immunosuppressive TME were investigated. It is discovered that during cholestasis, neutrophils are significantly recruited and in turn impair the function of CD8^+^ T cells, which eventually leads to an immunosuppressive TME and augmented LM. Controlling neutrophil phenotype switching by influencing the synthesis of BAs may offer new opportunities for therapeutic strategies in LM under cholestasis.

## Methods

### Patient samples

All CRLM patients enrolled in the study were admitted to the Oncology Department of Nanfang Hospital, Guangdong, P.R. China during 2017-2021. A total of 37 samples (13 cholestatic and 24 non-cholestatic samples) were collected from 11 cholestatic and 19 non-cholestatic patients. The basal information of patients (demography) presenting cholestasis and disease features were provided in Supplementary Table S1. Six paired non-cholestatic and cholestatic samples were collected from five patients who had developed from non-cholestatic to cholestatic conditions during the disease. The paraffin-embedded tumor samples were obtained from seven patients with cholestasis and eight patients without cholestasis. Among the seven patients with cholestasis, two presented with cholestatic-associated CRLM before chemotherapy, whereas the remaining five developed cholestasis during disease progression. The CRC liver metastatic samples were collected from the patients using percutaneous transhepatic puncture biopsy for pathological examination, immunohistochemistry, immunofluorescence, and RNA-sequencing (RNA-Seq) studies. The therapeutic effects were evaluated according to the Response Evaluation Criteria in Solid Tumors (RECIST) version 1.1 [15]. The criteria to determine cholestasis included any of the following: 1) direct bilirubin (DBIL) > 13 μmol/L; 2) total bilirubin (TBIL) > 22.24 μmol/L; and 3) total BA > 10.0 μmol/L. As for the Kaplan Meier analysis of progression-free survival (PFS), 2 patients were excluded due to lack of survival data.

The use of human CRLM tumor tissues, and blood samples of healthy donors and mCRC patients with or without cholestasis was approved by the institutional review board at Southern Medical University Nanfang Hospital and complied with all relevant ethical regulations. Written informed consent was obtained from all patients.

### Animals

Female C57BL/6 mice of 4 to 6 weeks old were purchased from Guangdong Animal Center.

### Cholestasis model

To establish the extrahepatic cholestasis model, the bile duct ligation (BDL) surgery was performed as described [16]. Briefly, mice were first anesthetized. Then, a median abdominal incision was cut and the common bile duct was identified. The duct was dissected carefully and doubly ligated with 7–0 Prolene (Ethicon, Somerville, NJ). In the sham operation group, the duct was dissected without ligation. The abdominal incision was closed in two-layer sand wiped with alcohol swab after surgery. The mice were kept warm until recovery and treated as indicated [17].

For the intrahepatic cholestasis model, the mice were intragastrically administered 75 mg/kg of α-naphthylisothiocyanate (ANIT) dissolved in 100 μL olive oil for only once [18].

### Liver metastasis model

To achieve LM in vivo, 5 × 10^5^ (50 μL) MC38 or Panc02 cells were injected into the spleen or 2.5 × 10^5^ (50 μL) cells were directly injected into the liver capsule per mouse. The tumor cell inoculation was performed two days later, two days in advance, or simultaneously with BDL surgery, as indicated in the different models.

## Neutrophil depletion, obeticholic acid (OCA) treatment, and anti-PD1 treatment

To achieve systemic neutrophil depletion, the mice were intraperitoneally injected with anti-Ly6G antibody (first dose 400 μg on day 0, the rest doses 100 μg on day 1, 2, 4, and 6). To alleviate cholestasis, OCA was intragastrically administered to mice at a dose of 30 mg/kg every day. For anti-PD1 treatment, mice were intraperitoneally injected with 200 μg anti-PD-1 antibody every three days for three times before being sacrificed.

### Cell lines and culture

The murine colon cancer cell line MC38, murine pancreatic cancer cell line Panc02, and human colon cancer cell line DLD1were purchased from ATCC (Manassas, VA, USA). DLD1 were cultured with RPMI 1640 supplemented with 10% fetal bovine serum (FBS). MC38 and Panc02 were cultured with DMEM supplemented with 10% FBS. Cell line authentication and mycoplasma testing were routinely performed for all cell lines.

### Isolation and culture of mouse hepatic cells

Hepatocytes were isolated as described [19]. Briefly, the liver was perfused with solution I (1 L HANKS solution I supplemented with Heparin sodium 0.1g) and Solution II (50 mL DMEM supplemented with CaCl2·H2O 0.029g and Collagenase IV 0.025g) for 5 min. Then the liver was grinded and filtered through a 70 μm mesh nylon strainer and later centrifuged at 50g/ 2 min, 4°C for 2 times for optimal hepatic cell-purification. Hepatic cells were seeded into a 6-well plate precoated with Collagen IV at a density of 2 × 10^6^. Culture medium contained 10% FBS, 100 nM dexamethasone, 2 mM glutamine and 1% penicillin/streptomycin solution. After 4 h of adhesion, unattached cells and medium were removed and replaced with fresh culture medium. The remaining adherent cells were hepatic cells.

Primary hepatocytes were stimulated with 200 μM Taurocholic acid (TCA) or 200 μM Glycocholic acid (GCA) for 24 h.

### Isolation of mouse neutrophils and T cells

To collect mouse neutrophils, bone marrow cells were flushed from femur and tibia with PBS. Cell suspension was removed of clumps and centrifuged with neutrophil separating medium and then purified with EasySep mouse neutrophil enrichment kit following manufacturer’s protocol.

To collect mouse T cells, isolated spleens were grinded on a 70 μm mesh nylon strainer and washed with phosphate-buffered saline (PBS). Cell suspension was enriched via murine lymphocyte isolation medium the murine CD8^+^ T cells specific magnetic beads according to manufacturer’s procedures.

### Isolation of human neutrophils and T cells

A total of 40 mL of peripheral blood was collected from healthy donors. Human neutrophil separating medium and human CD8^+^ T cells magnetic beads were used for purification.

### Co-stimulation and culture of T cells

Murine and human T cells were cultured with RPMI 1640 supplemented with 10% fetal bovine plasma, 30 U/mL interleukin-2 (IL-2) and 200 μM L-glutamine. Mice T cells were activated with CD3/CD28, while human T cells were activated with CD3/CD28 for 3 days before further assays.

### Bile acids or p38 inhibitor stimulation

Bile acids added into culture medium respectively for 24 h of stimulation: TCA 200 μM, GCA 200 μM, Tauro-β-muricholic acid (Tβ-MCA) 200 μM. The p38 Mitogen-activated protein kinase (p38 MAPK) inhibitor SB203580 and SB202190 were added into medium of certain groups of mice and human neutrophils or lymphocytes in a concentration of 50 nM.

### Coculture of neutrophils and T cells

CD8^+^ T cells were primed using CD3/CD28 for 3 days. Neutrophils were stimulated with bile acids or p38 MAPK inhibitor SB203580/SB202190 for 12 h before use. Then the primed-CD8^+^T cells and bile acids-stimulated neutrophils were removed of previous culture medium before being cocultured in the same petri dish for 24 h using T cell culture medium (supplemented with IL-2 and L-glutamine).

### Cell killing assay of T cells/ Tumor cell apoptosis assay

MC38 and DLD1 were pre-seeded in a 6-well plate at a density of 2.5×10^5^ cells/well one day before coculturing with murine or human CD8^+^ T cells. After neutrophil-CD8^+^ T cell coculture for 24h, CD8^+^ T cells were isolated from neutrophils using magnetic beads as described above. Then CD8^+^ T cells counted and diluted to reach a number 4 times more than the seeded tumor cells (CD8^+^ T cells: tumor cells = 4:1). CD8^+^ T cells were seeded in the wells with tumor cells to achieve direct coculture for 24 h. At last, all cells were collected and dyed with Annexin V/ PI for flow cytometry.

### RNA sequencing analysis (RNA-Seq)

The liver metastases tissues of CRC patients, liver metastases tissue of Sham/BDL mice, and neutrophils stimulated with different BAs were sequenced and analyzed (Geneplus-Beijing Co. Ltd., Beijing, P. R. China for patient samples; Guangzhou Gene Denovo Biotechnology Co. Ltd., Guangdong, P. R. China for mouse liver metastatic tissues, and Wuhan Seqhealth Co., Ltd., Hubei, P. R. China for stimulated neutrophils). Raw sequencing data was first filtered by Trimmomatic (version 0.36). Reads mapped to the exon regions of each gene were counted by featureCounts (Bioconductor) before RPKM was calculated. Genes differentially expressed between groups were identified using the edgeR package (version 3.12.1). A *p*-value cutoff of 0.05 and Fold-change cutoff of 2 were used to judge the statistical significance of gene expression differences. Gene ontology (GO) analysis and Kyoto encyclopedia of genes and genomes (KEGG) enrichment analysis for differentially expressed genes were both implemented by KOBAS software (version 2.1.1) with a *P*-value cutoff of 0.05.

### Liquid Chromatograph Mass Spectrometry (LC/MS)

Liver and blood bile acids were detected by LC/MS. The whole liver metastatic tumor and 450 μL peripheral blood of each mouse were collected. Mouse peripheral blood was centrifuged at 450 g for 10 min at 4 °C. Plasma were further extracted and performed with LC/MS analysis as described [20] by Metware Biotechnology Co., Ltd. (Wuhan, China).

### Flow cytometry and Fluorescence activated Cell Sorting (FACS)

Single-cell suspension was obtained from mice liver or human peripheral blood as described above. Cells were dyed with flow cytometric antibodies for neutrophils (mouse: CD45^+^Ly6G^+^CD11b^+^, human: CD45^+^CD66b^+^CD11b^+^), effector memory T cells (CD4^+^CD44^+^CD62L^-^) and CD8^+^ T cells. CD8^+^T cells were labeled with carboxyfluorescein diacetate succinimidyl ester (CFSE) for proliferation investigation. Flowjo VX app was used to analyze the results. Antibodies were listed in supplementary Table S2.

### Immunofluorescence

Tissues sections obtained from paraffin-embedded mouse and human CRLM tissues were stained with antibodies as indicated.

Neutrophils were planted on confocal dishes and cultured before being fixed and penetrated using 10% Triton-X. Then cells were stained with antibodies as indicated. Antibodies were listed in supplementary Table S2. All immunofluorescent samples were observed and photographed using Olympus confocal microscope.

The immunofluorescent score of tissue-slide samples considered only the number of immune cells marked by antibodies with immunofluorescent labels. Five random fields were photographed and the number of fluorescent cells per field were counted. The immunofluorescent score for neutrophils considered the ratio of mean fluorescent intensity (MFI) of neutrophils per field. To be specific, the MFI of iNOS, Arg1, Ly6G or CD66b were quantified by Image J 8.0. The final immunofluorescent score of iNOS in murine neutrophils = iNOS MFI/Ly6G MFI; iNOS MFI/CD66b MFI in human neutrophils. And so on for Arg1.

### Immunohistochemistry, H&E staining and sirius red staining

For immunohistochemistry, paraffin-embedded tissue slides of mice liver metastatic tumors and subcutaneous tumors of different groups were dewaxed, and performed with antigen retrieval according to standard procedures. Tissues were stained with antibodies for 16 h before being washed and incubated with Goat HRP antibodies. At last, tissues were stained with DAB and hematoxylin before being washed, dehydrated and sealed in neutral balsam. The immunohistochemical staining results were mean scores considering only the number of immune cells marked by 3,3’-diaminobenzidine (DAB). Under different magnification, 5 random fields were photographed and the number of brown-marked cells per field were counted.

H&E staining of tumor tissue was performed as described. Briefly, tissue slides were fully dewaxed then washed with PBS. Then, tissues slides were stained with hematoxylin and eosin (H&E). At last, they were dehydrated and sealed.

Sirius Red staining of tumor tissue for fibrous tissue was performed following manufacturer’s instructions.

### Enzyme-linked immune sorbent assay (ELISA)

ELISA assays were performed to investigate the chemokines and cytokines expression of neutrophils and hepatocytes following standard manufacturer’s procedures.

### Western blot

Proteins of neutrophils stimulated with bile acids and p38 MAPK inhibitor SB203580/ SB202190 were extracted using protein extraction kit following manufacturer’s protocol. Western blot was performed according to standardized protocol as described. Antigen were listed in supplementary Table S2.

### Quantitative Real-time Polymerase Chain Reaction (qPCR)

Ribonucleic acid (RNA) of neutrophils stimulated with bile acids and p38 MAPK inhibitor SB203580/SB202190 were extracted using RNA extraction kit following manufacturer’s instructions. RNA was reversed into cDNA before 40 cycles of amplification and quantification. Relative gene expression levels were quantified based on the cycle threshold (Ct) values normalized to the reference gene β-actin or glyceraldehyde-3-phosphate dehydrogenase (GAPDH). Primer sequences were listed in supplementary Table S3.

### Patient clusters identifying

The colon cancer data from the Cancer Genome Atlas (TCGA) were downloaded from the UCSC Xena browser (GDC hub: https://gdc.xenahubs.net). Updated clinical data and sample information of TCGA-COAD samples were obtained from UCSC Xena browser. The RNA-sequencing data (FPKM values) of the TCGA-COAD were transformed into transcripts per kilobase million (TPM) values before further analysis. The differentially expressed genes (DEGs) of TCGA-COAD were analyzed using the “DESeq2” R package [21]. Then DEGs were performed with univariate Cox survival analysis, genes that are significantly correlated with the survival of TCGA-COAD (*p* < 0.05) were selected and referred to as “survival related DEGs” for further analysis.

Bile acid metabolism genes were collected from the MSigDB database [22], p38 MAPK signaling genes were collected from GeneCards website (https://www.genecards.org/), tumor-associated neutrophil genes were provided by Zhang etl [23]. The three gene sets were first merge as one gene set. The merged gene set was then intersected with the “survival related DEGs” and generated a final list of 8 genes (Supplementary Table S4). Then, patients of TCGA-COAD were performed with unsupervised clustering methods (K-means) [24] to identify two distinct patient clusters. The two groups of patients “BPNC 1” and “BPNC 2” were performed with Kaplan–Meier survival analysis. The ImmuneScore is calculated as described [25].

### Bioinformatics analysis

All RNA-Seq count data were analyzed using R 4.1.0. The gene expression matrix (normalized to TPM and FPKM) was processed using the sva R package [26] to account for the batch effects. The R package DESeq2 [21] was used for all DEGs analyses. The Benjamini–Hochberg [27] correction was applied to convert the *P*-values to adjusted *P*-values. Gene set enrichment analysis (GSEA) [28] was performed using the R package clusterProfiler [29]. The immune cell infiltration was analyzed using the R package IOBR, which consists of the CIBERSORT TME deconvolution method (https://github.com/IOBR/IOBR) [30].

### Statistics

Data analysis was performed using the GraphPad Prism software (version 8.0). For animal studies, the unpaired Student’s t-test was used to compare the mean between the two groups, and the Kaplan–Meier test was used to determine the survival rate. The correlation coefficients were computed using Spearman’s correlation and distance correlation analyses. For comparisons of more than two groups, one-way ANOVA was used for parametric methods. For human subjects, statistics comparing different parameters were determined using the Chi-square test, whereas the progression-free survival was determined using the Kaplan–Meier analysis. The differences were considered statistically significant when the *P*-values were determined to be less than 0.05 for two-tailed *t-test*.

## Results

### Cholestasis contributes to liver metastasis of colorectal cancer

**Fig. 1:**
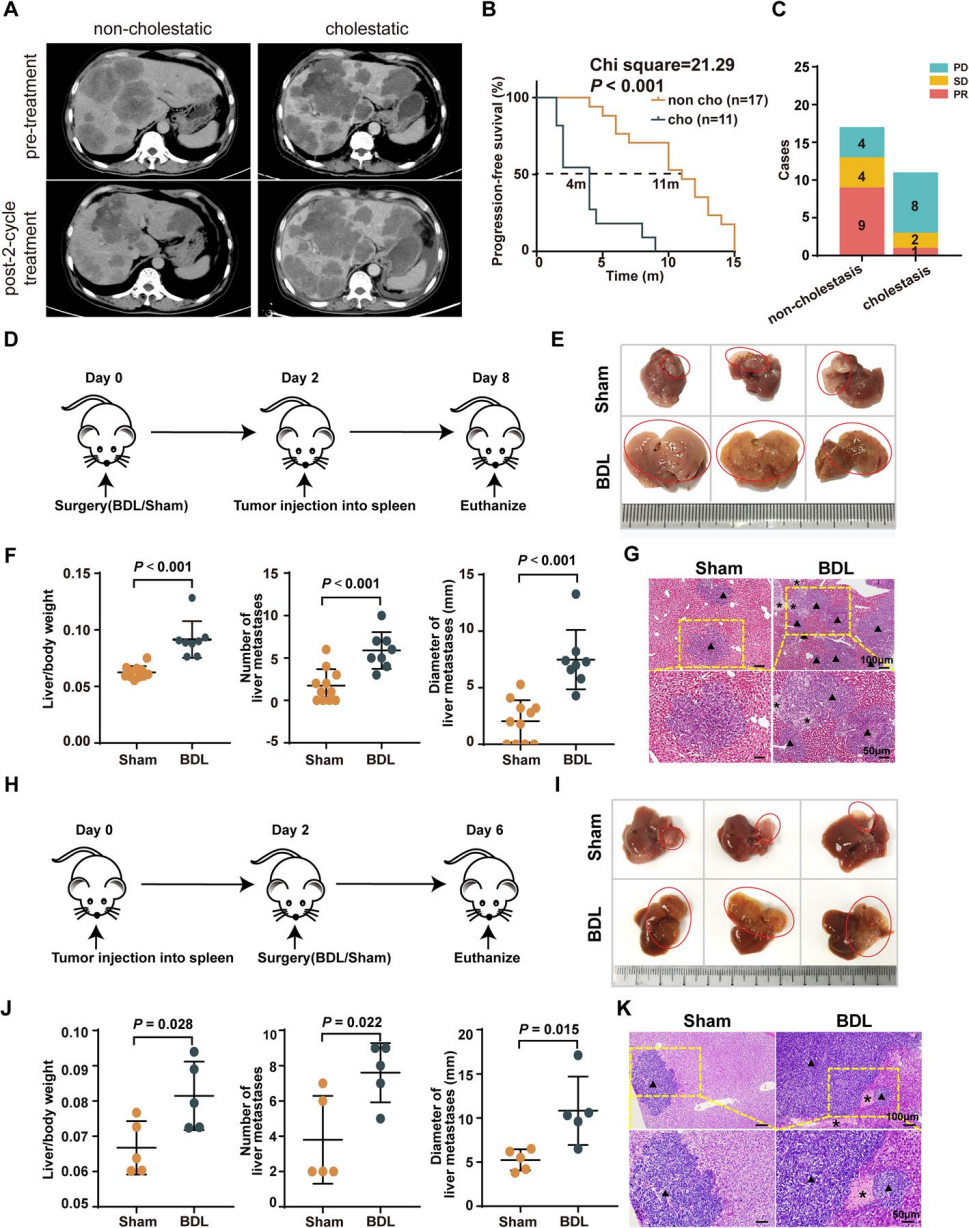
Biliary obstruction contributed to liver metastasis of colorectal cancer. **A** Representative CT images of non-cholestatic and cholestatic colorectal cancer liver metastasis patients. **B** Kaplan–Meier analysis of the progression-free survival of patients with non-cholestatic (*n* = 17) and cholestatic (*n* = 11) LM. Log-rank test. **C** Cases of first-line therapeutic response of CRLM patients. PR, partial response; SD, stable disease; PD, progressive disease. **D** Female C57BL/6 mice were subjected to Sham/BDL surgery before being administered an intrasplenic injection of MC38 tumor cells to form LM, and were euthanized on day 8 (BT model). **E** Representative images of LM described in panel (**D**). **F** Quantitation of liver/body weight ratio, tumor nodules, and diameters of LM of the BT model mice (*n* = 11 and 8 for Sham and BDL groups, respectively). **G** Representative images of the HE-staining of LM sections described in panel (**D**). **H** C57BL/6 mice were administered an intrasplenic injection of MC38 tumor cells to form LM before receiving the Sham/BDL surgery, and were euthanized described in panel (**H**). Data represent mean ± SD of 3 biologically independent experiments (Two-tailed t test). Sham, sham surgery; BDL, bile duct ligation surgery; asterisk, liver injury; arrowheads, tumor metastases; asterisks, apoptotic lesionson day 6 (TB model). **I** Representative images of LM described in (**H**). **J** Quantitation of liver/body weight ratio, tumor nodules, and diameters of LM of the TB model mice (*n* = 5 per group). **K** Representative images of the HE-staining of LM sections

To explore the potential correlation between cholestasis and colorectal cancer liver metastasis, we screened the computed tomography of 30 patients diagnosed with colorectal cancer liver metastasis (CRLM) and classified them as non-cholestatic and cholestatic according to serum level of total bile acids (TBA), total bilirubin (TBIL), direct bilirubin (DBIL), alkaline phosphatase (ALK), alanine aminotransferase (ALT), and aspartate aminotransferase (AST). The clinical and demographic characteristics of patients diagnosed with CRLM is listed in supplementary Table S1. Patients with cholestasis were observed with slightly more frequent KRAS mutation, higher T stage and less primary tumor resection, indicating a more malignant biological behavior of the tumor, which further contributed to a shorter survival (Supplementary Table S1). Consistent with the clinical characteristics, CRLM with cholestasis developed aggressively and responded poorly to anti-tumor therapy (Fig. 1A), with a decreased median PFS (11 vs. 4 months, *P* < 0.001, Fig. 1B), and poorer treatment response (52.9% vs. 9%, Fig. 1C). These results suggest that cholestasis mediated CRLM progression, while the underlying mechanisms remain unclear.

In order to verify the role of cholestasis on CRLM progression. Cholestasis CRLM mice model was further constructed by BDL surgery (extrahepatic cholestasis) and ANIT feeding (intrahepatic cholestasis) according to the previous study [16]. Cholestasis caused by disease-associated obstruction of the common bile duct is defined as extrahepatic cholestasis, whose feature could be recapitulated by BDL surgery (extrahepatic cholestasis, Fig. 1D, H). Cholestasis resulted from damage or obstruction of various hepatic duct within the liver is referred to as intrahepatic cholestasis, which could be established via oral gavage of ANIT, a chemical that induces acute hepatic duct damage and necrosis of hepatocytes (intrahepatic cholestasis, Supplementary Fig. S2A, S2D) [18]. These two models represent the different clinical situation in patients with cholestasis. Both BDL- and ANIT-subjected mice were observed with decreased vitality, poor appetite and survival, increased plasma concentrations of ALT, AST, TBIL, and DBIL (Supplementary Fig. S1A-1B). Furthermore, accumulation of collagen in liver tissues, especially in areas adjacent to the hepatic duct, was observed 8 days post-BDL surgery, which is consistent with previous findings that chronic cholestasis can induce fibrosis and cirrhosis (Supplementary Fig. S1C). The metastatic tumor in the liver was verified by CK19 immunochemical staining (Supplementary Fig. S1D). Therefore, these results demonstrated that extrahepatic and intrahepatic cholestasis model was successfully established.

To study the interaction between cholestasis and LM, murine colon cancer cells MC38 were injected into the spleen 2 days after Sham/BDL (BT model, Fig. 1D). Mice were euthanized and organs were collected at the 8th day (Fig. 1D). It was discovered that BDL-subjected mice had larger metastatic tumors (Fig. 1E, F) than those of the Sham mice. Besides, HE staining (Fig. 1G) and CK19 immunochemical staining which refers to tumor lesions (Supplementary Fig. S1D) further confirmed the pro-tumor role of BDL at pathological level. Moreover, liver necrosis and ballooning degeneration (Fig. 1G) were more frequently detected in the tissues of BDL-subjected mice than in those of Sham mice, which was consistent with cholestatic hepatic injury. On the other hand, ANIT-treatment model prior to MC38 inoculation (Supplementary Fig. S2A, AT model) was established. In consistent with BDL surgery, ANIT-induced cholestasis enhanced the liver metastasis of CRC (Supplementary Fig. S2B–2C). These results implied that cholestasis induced either by BDL surgery or ANIT oral gavage is a pro-tumor factor towards colorectal cancer liver metastasis.

Cholestasis usually progresses along with the development of liver tumors or metastatic lymph nodes, which causes detortion of intrahepatic biliary ducts, as well as obstruction of the common bile duct, inhibiting normal bile flow. Therefore, to evaluate whether the initiation time point of cholestasis had an impact on the development of LM in CRC, several additional tumor models with different obstruction locations and initiation time points were established. Mice were first injected with MC38 cells before performed with Sham/BDL surgery (Fig. 1H, TB model). In consistent with the BT model, the results of TB model showed that BDL surgery enlarged the diameter of metastases of MC38-liver tumor, increased the liver/body weight ratio and total metastases nodes (Fig. 1I-K). In consistent with the TB model, ANIT oral gavage two days after MC38 inoculation (Supplementary Fig. S2D, TA model) also enlarged the diameters and weight of metastatic lesions significantly (Supplementary Fig. S2E-2F). These results indicate that, regardless of initiating before or after the formation of liver metastasis, cholestasis could accelerate the progression of liver metastasis. Therefore, we mainly focused on the model in which cholestasis was established prior to LM formation, the BT model.

To study the impact of cholestasis on the variety of liver metastases, we established orthotopic inoculation of colon cancer cell MC38 on the liver (liver capsule injection model). MC38 cells were injected into mice’s liver capsule and observed after 7 days (Supplementary Fig. S2G). In consistent with the previous results, MC38 tumor sizes were larger in BDL mice (Supplementary Fig. S2H-2I). To study if cholestasis could impact liver metastases of other pathological characteristics, the murine pancreatic carcinoma cell line Panc02 were injected into the liver capsule of mice. We observed similar results (Supplementary Fig. S2J-2L).

Together, these results indicated that regardless of the initial time of cholestasis, tumor types or metastatic routes, cholestasis accelerates the development of liver metastasis.

### Microenvironment of CRC LM during cholestasis develops into immunosuppressive TME

**Fig. 2:**
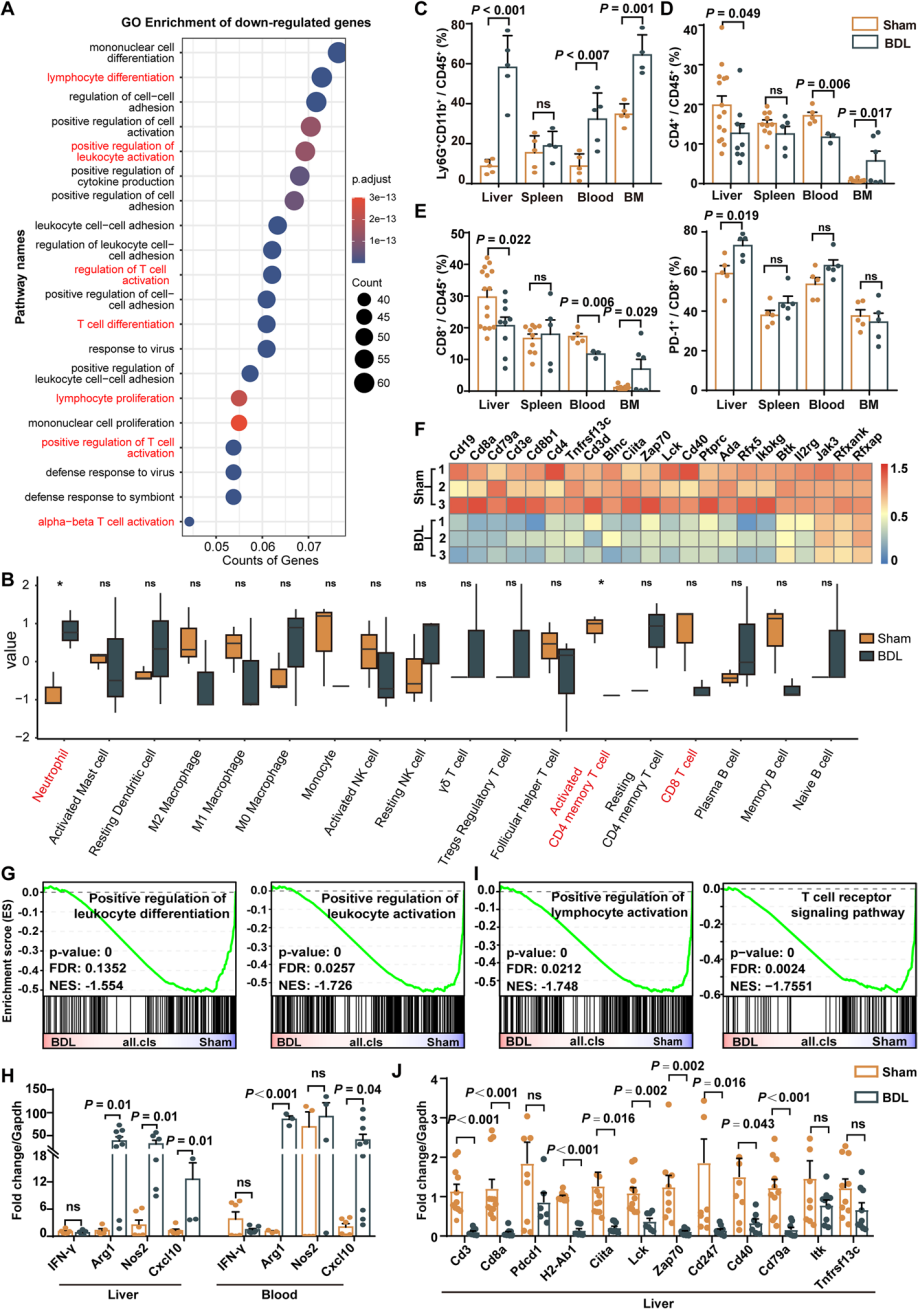
Cholestatic MC38-liver metastasis has an immunosuppressive microenvironment associated with neutrophil accumulation and T cell exclusion. **A** GO biological process analyses of DEGs of the LM of Sham/BDL MC38-bearing BT model mice. **B** CIBERSORT analysis of TME cells in the LM of Sham/BDL mice. The thick lines represent the median values. The bottom and top of the boxes represent the 25th and 75th percentiles (interquartile range), respectively. The whiskers encompass 1.5 times the interquartile range. **C**-**E** Flow cytometric analysis of neutrophils (**C**), total CD4^+^ T cells (**D**), total CD8^+^ T cells and PD-1^+^CD8^+^ T cells (**E**) of MC38-bearing BT model mice. F, Heat map of lymphocyte activation related genes between Sham and BDL mice. **G**-**H** GSEA result of the positive regulation of leukocyte differentiation pathway (**G**) and related mRNA expression in LM tissues (**H**). **I, J** GSEA result of the leukocyte activation pathway and T cell receptor signaling pathway (**I**) and related mRNA expression in LM tissues (**J**). Data represent mean ± SEM of 3 biologically independent experiments (Two-tailed t test). ns, no significance; Sham, sham surgery; BDL, bile duct ligation surgery

To gain insight into the mechanisms underlying cholestasis-associated CRC LM, the MC38-induced metastatic tumors from the BT model were analyzed through RNA-sequencing. The GO and KEGG pathway analyses indicated that the downregulated genes were mostly associated with the positive regulation of immune processes such as lymphocyte differentiation, proliferation, and activation (Fig. 2A, Supplementary Fig. S3A). Immune activation-associated genes and signaling pathways including interferon α (IFN-α) and interferon γ (IFN-γ), and positive regulation of T cell activation were downregulated in the BDL-subjected group (Supplementary Fig. S3B). Moreover, CIBERSORT analysis demonstrated that the infiltration of effector CD4^+^ memory T cells and CD8^+^ T cells, which contribute to the immune-killing effect [7], was significantly decreased, while infiltration of neutrophils was remarkably increased in the CRC LM of BDL-subjected mice (Fig. 2B).

Flow cytometry has confirmed that in the liver and blood of BDL-subjected mice, there was increased proportion of Ly6G^+^CD11b^+^ neutrophils (Fig. 2C and Supplementary Fig. S3C). Total CD4^+^ T cells (Fig. 2D), effector memory CD4^+^ T cells (CD44^+^CD62L^-^ CD4^+^, Supplementary Fig. S3E-3F) were significantly downregulated in the liver and blood of BDL mice, as compared to that of the Sham mice. Moreover, the proportion of T lymphocytes were significantly decreased, including total CD8^+^ T cells (Fig. 2E and Supplementary Fig. S3D) in the liver, blood, and bone marrow. Remarkably, the PD-1 expression of CD8^+^ T cells was significantly increased in cholestatic group (Fig. 2E and Supplementary Fig. S3E), indicating enhanced exhaustion of CD8^+^ T cells [31]. Myeloperoxidase (MPO), a heme-containing peroxidase, is expressed by neutrophils to kill microbes. MPO is a biomarker widely used to indicate neutrophils in many studies [32]. However, in the bone marrow of the BDL group, there was an increase in the proportion of CD8^+^ and CD4^+^ T cells, which might due to the negative feedback towards immune suppression in the liver and blood (Fig. 2D, E, Supplementary Fig. S3F). Immunostaining also confirmed the results (Supplementary Fig. 2G). In consistent with the CIBERSORT result (Fig. 2B), no significant difference was observed in the infiltration of natural killer cells, dendritic cells, regulatory T cells, or macrophages in the CRLM between two groups (Supplementary Fig. S3H).

Similar alterations of immune infiltration were also observed in the TB model (Supplementary Fig. S4A–4D), the intrahepatic cholestasis model (AT model, Supplementary Fig. S4E-4G), as well as in the liver capsule injection models with MC38 and Panc02 cells via flow cytometry assay (Supplementary Fig. S4H-4I). The immunohistochemistry staining of liver tumor tissue section also demonstrated similar immune infiltration results (Supplementary Fig. S4J). These results suggest that increased neutrophils infiltration and T cells exclusion and exhaustion were the major changes in cholestatic LM microenvironment.

The GSEA analysis demonstrated that leukocyte differentiation- and activation-related genes were significantly decreased in BDL group (Fig. 2F-G), suggesting the neutrophils might exhibited an immunosuppressive phenotype in cholestasis TME. Therefore, marker genes related to neutrophils activation and immunosuppression were further detected. In BDL-subjected mice, neutrophils isolated from the liver and peripheral blood had increased gene expression of Arg1, NOS2, and CXCL10, which but no changes of IFN-γ (Fig. 2H). As shown in Fig. 2H, arginase 1 (Arg1) and nitric oxide synthase 2 (NOS2), which was contributed to neutrophils immunosuppressive by inhibiting T cells activation [33, 34], were significantly upregulated in cholestasis liver metastasis. However, no change was indicated in IFN-γ expression, which was reported to mediate liver inflammation (Fig. 2H) [35]. In addition, C-X-C motif chemokine ligand 10 (CXCL10) was also elevated in cholestasis group (Fig. 2H). As reported, CXCL10 can recruit both immunoactive cells such as CD8^+^T cells and CD4^+^T cells [36], and immuosuprresive cells like MDSCs into the tumor microenvironment [37]. Due to the two-sided effect of CXCL10 in recruiting both cytotoxic cells and immunosuppressive cells, CXCL10 might have indicated heterogenous phenotypes of neutrophils in the cholestatic tumor microenvironment. Meanwhile, the GSEA of the DEGs showed that lymphocyte activation, especially the T cell activation pathway, was remarkably inhibited in BDL-subjected mice (Fig. 2I), which was consistent with the decreased expression of TCR-activating genes (Lck, Zap70, CD247) on mRNA levels (Fig. 2J). Lymphocyte related genes (CD3, CD8), B cell receptor related genes (CD40, CD79a), major histocompatibility complex (MHC) related genes (H2-Ab1, Ciita) were significantly downregulated in the tumor tissues of BDL mice. However, interleukin-2(IL-2) inducible T cell kinase gene (Itk), TNF Receptor Superfamily Member 13C (Tnfrsf13c), and Programmed Cell Death 1 gene (Pdcd1) showed no significant difference between BDL and Sham mice (Fig. 2J). These results indicated that cholestasis significantly inhibited the differentiation and activation, as well as antigen-presenting function of leukocytes, which may further induce the dysfunction of T cells and tumor progression.

Together, these results strongly indicate that cholestasis leads to an immunosuppressive microenvironment that further enhances the progression of LM in CRC.

### Neutrophils contribute to the cholestasis-induced LM immune evasion in CRC

Next, we explored the underlying mechanisms of the alteration of TME induced by cholestasis. Neutrophils play critical and complex roles in cancer immunity and metastases. We speculated that the infiltrating neutrophils led to the formation of an immunosuppressive microenvironment [38].

To understand the increased infiltration of neutrophils in the liver and peripheral blood, the GSEA analysis was performed. Compared to Sham mice, chemokine-chemokine receptor interaction pathway was activated in BDL mice (Supplementary Fig. S5A). Cholestasis induce liver injury and inflammation, which leads to increased level of chemokines and cytokines [39]. The C-X-C chemokine receptor 1 and 2 (CXCR1 and CXCR2), two of the most common chemokines and their C- X-C motif ligands (CXCL), such as CXCL1-3, CXCL5-7, and CXCL8 have been documented to drive the accumulation of neutrophils [40]. The genes related to the cytokine-cytokine receptor interactions were differentially enriched in Sham/BDL-subjected mice. CXCL2 and CXCL5 were the only two chemokine related genes whose RNA-seq (Supplementary Fig.S5B) results that were consistent with our qPCR results (Supplementary Fig. S5E), and the upregulation of CXCL2 and CXCL5 expression were the most significant, which was validated by qPCR analysis both in TB model and BT model (Supplementary Fig. S5C). In contrast, C-C motif chemokine ligand 2 (CCL2) also significant changed while with inconsistent results in BT and TB model (Supplementary Fig. S5B-S5C), therefore we focused on CXCL2 and CXCL5 in the following analysis. As indicated by the ELISA result, cholestatic liver also secreted more CXCL2 and CXLC5 to attract neutrophils (Supplementary Fig. S5D). Accordingly, chemokines can be produced and released by hepatocytes stimulated with BAs to recruit myeloid cells [16, 41, 42]. Therefore, we isolated the hepatocytes from normal mice and treated them with GCA or TCA *in vitro* clarify the producing of CXCL2 and CXCL5 in cholestasis liver. As a result, CXCL2 and CXCL5 was significantly increased in BAs stimulated hepatocytes both at mRNA and secreted protein lever in a time- and concentration-dependent manner (Supplementary Fig. S5E–5F). These results suggest that neutrophil recruitment during cholestasis is mediated by CXCL2 and CXCL5 derived from hepatocytes under the stimulation of excessive primary BAs.

It is crucial to learn about the initiation time of cholestasis and immune infiltration in order to verify their causal relation. Studies have shown that BDL surgery is able to cause acute liver inflammation, indicated by significantly upregulated ALT, AST, TBIL and DBIL levels within 72h hours [43, 44]. Furthermore, ANIT oral gavage of a dose of 70-75mg/kg is able to significantly increase the level of the above parameters within 48h [20]. Mice received BDL surgery or ANIT oral gavage are observed with jaundice within 48h, which is consistent with previous studies (Supplementary Fig. S6A). These results indicate that cholestasis occurred by the 48h post induction at the latest. In order to clarify the initiation time of immune infiltration, we have constructed BDL and ANIT mouse models and sacrificed in different time to examine neutrophils by flow cytometry. We clarified that neutrophil infiltration in the liver and accumulation in the blood were initiated 24-48h after implementation of either BDL surgery (Supplementary Fig. S6B-6C) or ANIT oral gavage (Supplementary Fig. S6D-6E), indicated by 2 foldchange increase of neutrophil percentage. This result is in line with the initiation of jaundice. Therefore, cholestasis and neutrophil accumulation in the liver is developed 24-48h post induction. The duration time for primary bile acid and neutrophil to impact on the tumor microenvironment before sacrificing the mice on the 8th day is adequate.

**Fig. 3:**
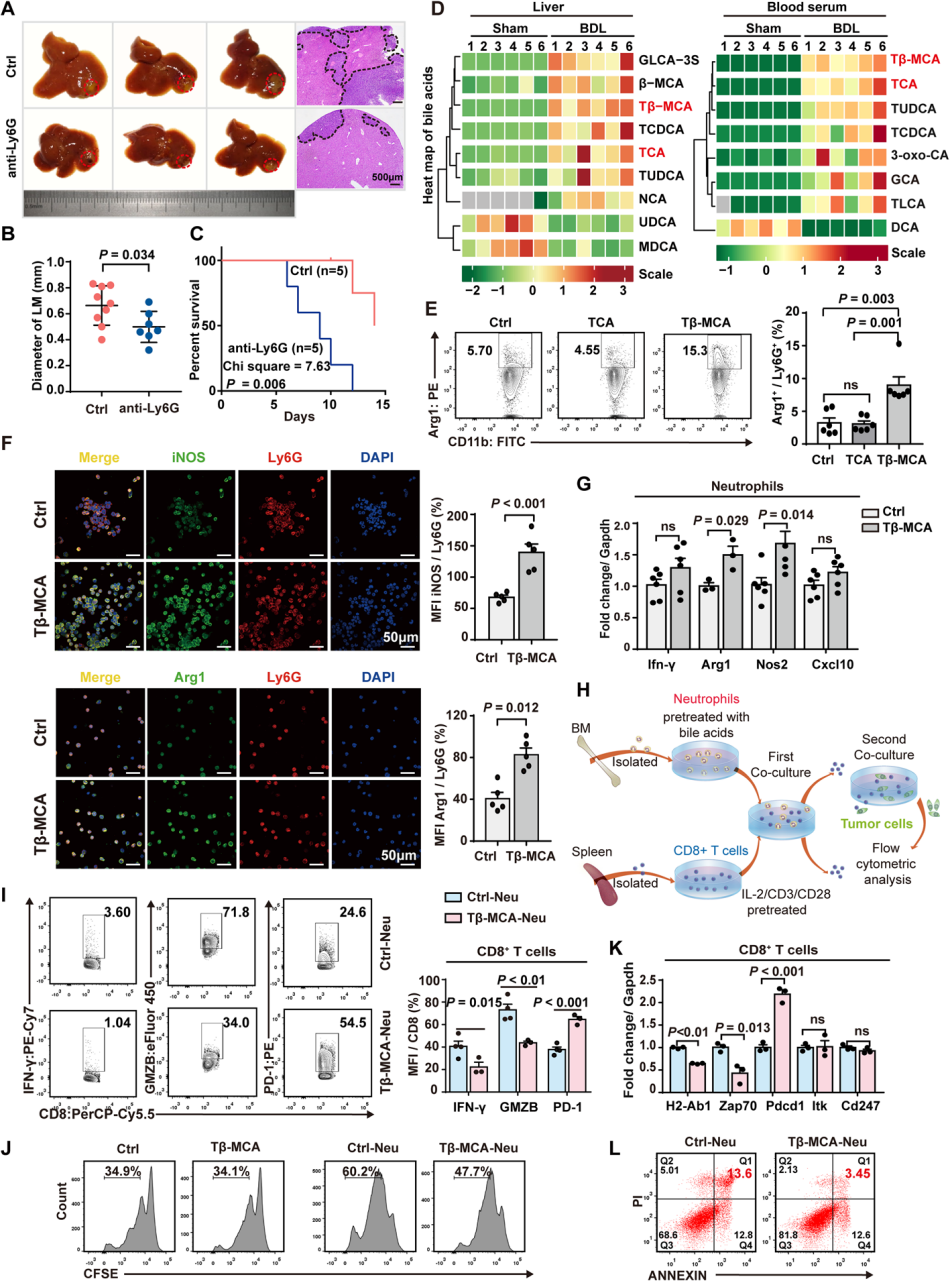
Primary bile acids contribute to the immunosuppressive phenotype of neutrophils. **A**, **B** C57BL/6 mice received BDL surgery and liver capsule injection of MC38 cells simultaneously on day 0, and a 400 μg anti-Ly6G i.p. injection on day 1, 2, 4, and 6, and were euthanized on day 8. Representative images of LM (**A**) and diameters of LM (**B**) of MC38-liver capsuled injected BDL mice after receiving anti-Ly6G treatment (*n* = 9 and 7 for the control and anti-Ly6G group, respectively). **C** Kaplan–Meier analysis of MC38-liver capsuled injected BDL mice with or without anti-Ly6G treatment (Log-rank test, *n* = 5 per group). **D** Bile acids from the liver (left) and serum (right) were analyzed by LC-MS. Hierarchical cluster analysis was performed on differential bile acid metabolites. TCA, Taurocholic acid; GCA, Glycocholic acid; Tβ-MCA, Tauro-β-muricholic acid; GLCA-3S, Glycolithocholic acid-3-sulfate; β-MCA, β-muricholic acid; TCDCA, Taurochenodeoxycholic acid; TUDCA, Tauroursodeoxycholic acid; NCA, Norcholic acid; UDCA, Ursodeoxycholic acid; MDCA, Murideoxycholic acid; 3-oxo-CA, 3-Oxocholic acid; TLCA, Taurolithocholic acid; DCA, Deoxycholic acid. **E** Flow cytometric analysis and quantitative results of Arg1 expression of neutrophils under 24h of TCA or Tβ-MCA stimulation (*n* = 6 per group). **F** Immunofluorescence images and quantification of expression of iNOS and Arg1 of neutrophils after Tβ-MCA stimulation. **G** The mRNA level of immunosuppressive genes of neutrophils after Tβ-MCA stimulation. **H** Schematic diagram of the co-culture system for neutrophils and lymphocytes. Isolated neutrophils were treated with bile acids and cocultured with CD3/CD28-primed CD8^+^ T cells for 24 h. Then, lymphocytes were isolated for the tumor cell killing assay and flow cytometry analysis. **I** Flow cytometric analysis and quantitation of IFN-γ, GZMB, and PD-1 expression of CD8^+^ T cells co-cultured with neutrophils pretreated with Tβ-MCA (*n* = 4 and 3 for the control and Tβ-MCA groups, respectively). GZMB, Grazyme-B. **J** Flow cytometric analysis of the proliferation of mouse CD8^+^ T cells co-cultured with Tβ-MCA-pretreated neutrophils. **K** The mRNA level of leukocyte differentiation-related genes of mouse CD8+ T cells after co-culturing with Tβ-MCA-pretreated neutrophils. Data represent mean ± SEM of 3 biologically independent experiments (Two-tailed t test). ns, no significance. **L** Flow cytometric analysis of apoptosis of MC38 cells induced by CD8+ T cells co-cultured with Tβ-MCA-pretreated neutrophils (*n* = 3 per group). Data represent mean ± SEM of 3 biologically independent experiments (Two-tailed t test). ns, no significance; Sham, sham surgery; BDL, bile duct ligation surgery

To verify the key role of neutrophils in the establishment of an immunosuppressive niche, anti-Ly6G antibody was administrated in BDL mice to deplete neutrophils (Supplementary Fig. S7A). As expected, anti-Ly6G administration attenuated LM progression in BDL mice (Fig. 3A, B). Furthermore, neutrophil depletion in turn restored the infiltration of total CD8^+^ T cells, total CD4^+^ T cells, and effector memory CD4^+^ T cells (Supplementary Fig. S7B-7E), together suggesting that liver metastasis induced by cholestasis mainly contributed to the neutrophils mediated immunosuppressive microenvironment. However, neutrophil depletion by anti-Ly6G antibody led to shorter survival of MC38-bearing BDL mice (Fig. 3C), probably due to loss of the protection of neutrophils against pathogens [40]. Therefore, cholestasis-associated neutrophil accumulation is related to the progression of liver metastasis. Even though neutrophil depletion partially restored the “immune-hot” microenvironment, it could not be applied in clinical practice.

### Primary bile acids participate in the neutrophil phenotypic transition and their recruitment in the CRC LM sites

Once neutrophils release from the bone marrow as immature cells, neutrophils mature in the blood and have their phenotype and function changed over time under various tumorigenic stimulation [45]. We hypothesized that the recruited neutrophils in the cholestatic liver metastasis elicited specific characteristics that eventually promoted the progression of LM in CRC patients with cholestasis. Therefore, we aimed to investigate the effect of cholestasis on neutrophil recruitment and phenotypic transition. Disordered BA metabolism is a hallmark characterized in cholestasis, showing elevated primary and secondary BA levels in both the liver and plasma [46]. Liquid chromatography and mass spectrometry (LC/MS) analysis showed that cholestasis significantly increased the level of taurocholic acid (TCA) and tauro-β-muricholic acid (Tβ-MCA), as well as other primary BAs including β-muricholic acid (β-MCA) and taurochenodeoxycholic acid (TCDCA), among which TCA and Tβ-MCA increased the most (Fig. 3D, Supplementary Fig. S8A–8B).

It is unknown whether alterations in the BA composition contribute to the recruitment and phenotypic transition of neutrophils. Thus, to investigate the role of BAs in the regulation of neutrophils, we intended to stimulate neutrophils with BA in vitro. According to reported studies, to mimic the in vivo conditions of cholestasis, it is recommended to apply a concentration of bile acids that ranges from 50 to 200 μM in vitro [17]. It was shown that 200 μM of GCA and Tβ-MCA serves best to stimulate neutrophils, detected by qPCR (Supplementary Fig. S8C). According to these results, we applied 200 μM concentration of all three bile acids in the proceeding in vitro assays that associate with neutrophils. Neutrophils isolated from the bone marrow of healthy mice were stimulated with TCA and Tβ-MCA and then performed with RNA-Seq analysis. According to the RNA-Seq results, Tβ-MCA, not TCA, significantly induced the differential gene expression of neutrophils (Supplementary Fig. S8D). GO analysis revealed that the genes related to the positive regulation of the immune response and activation were downregulated in the Tβ-MCA-treated neutrophils (Supplementary Fig. S8E). Meanwhile, *in vitro* stimulation of Tβ-MCA instead of TCA significantly increased the neutrophil expression of Arg1 and iNOS at both protein (Fig. 3E-F) and mRNA levels (Fig. 3G). Although CXCL10 was significantly increased in neutrophils in the liver metastases tumor in BT model mice (Fig. 2H), primary bile acid stimulation on neutrophils in vitro did not impact their expression level of CXCL10 (Fig. 3G, Supplementary Fig. S9C, S11I). Therefore, it was implied that the expression of CXCL10 was influenced by various factors other than the bile acids, which made it unsuitable for the indication of neutrophil phenotype. Furthermore, the co-culture of CD8^+^ T cells with Tβ-MCA-treated neutrophils for 24 h (Fig. 3H) resulted in inhibited proliferation of CD8^+^ T cells (Fig. 3J). After co-culture, CD8^+^ T cells were also observed with enhanced PD-1, and decreased IFN-γ and Grazyme B (GZMB) expression (F ig. 3I). H2-Ab1 belongs to the MHC-II family that assist antigen presentation, while Zap70 functions in the initial step of TCR-mediated signal transduction [47]. The downregulation of H2-Ab1 and Zap70 genes in BDL mouse liver tumors indicates the suppressed antigen presenting and lymphocyte function (Fig. 3K). Furthermore, Pdcd1 gene, also known as programming the Programmed cell death protein 1, is an immune-inhibitory receptor expressed in activated T cells to suppress its cytotoxic function. Increased expression of Pdcd1 indicates exhaustion of T cells and dysfunction of the anti-tumor effect (Fig. 3K). CD8^+^ T cells’ cytotoxic effect on MC38 cells was also impaired (Fig. 3L). However, direct stimulation with Tβ-MCA did not exert such an effect on T cell proliferation (Fig. 3J).

Glycocholic acid (GCA) is the most abundant primary BA in patients with LM and cholestasis [48]. Similar results were noted in human neutrophils treated with GCA, displaying higher expression of Arg1 (Supplementary Fig. S9A-9B). Human T cells co-cultured with GCA-treated neutrophils were also suppressed in expression of TCR markers, proliferation, and cytotoxicity, while exhaustion marker PD-1 expression increases (Supplementary Fig. S9C-9G). These results thus showed that BAs induce phenotypic changes in neutrophils, resulting in the inhibition of lymphocyte activation and enhancement of the immunosuppressive microenvironment.

### Bile acids mediated neutrophils transition by p38/MAPK signaling

**Fig. 4:**
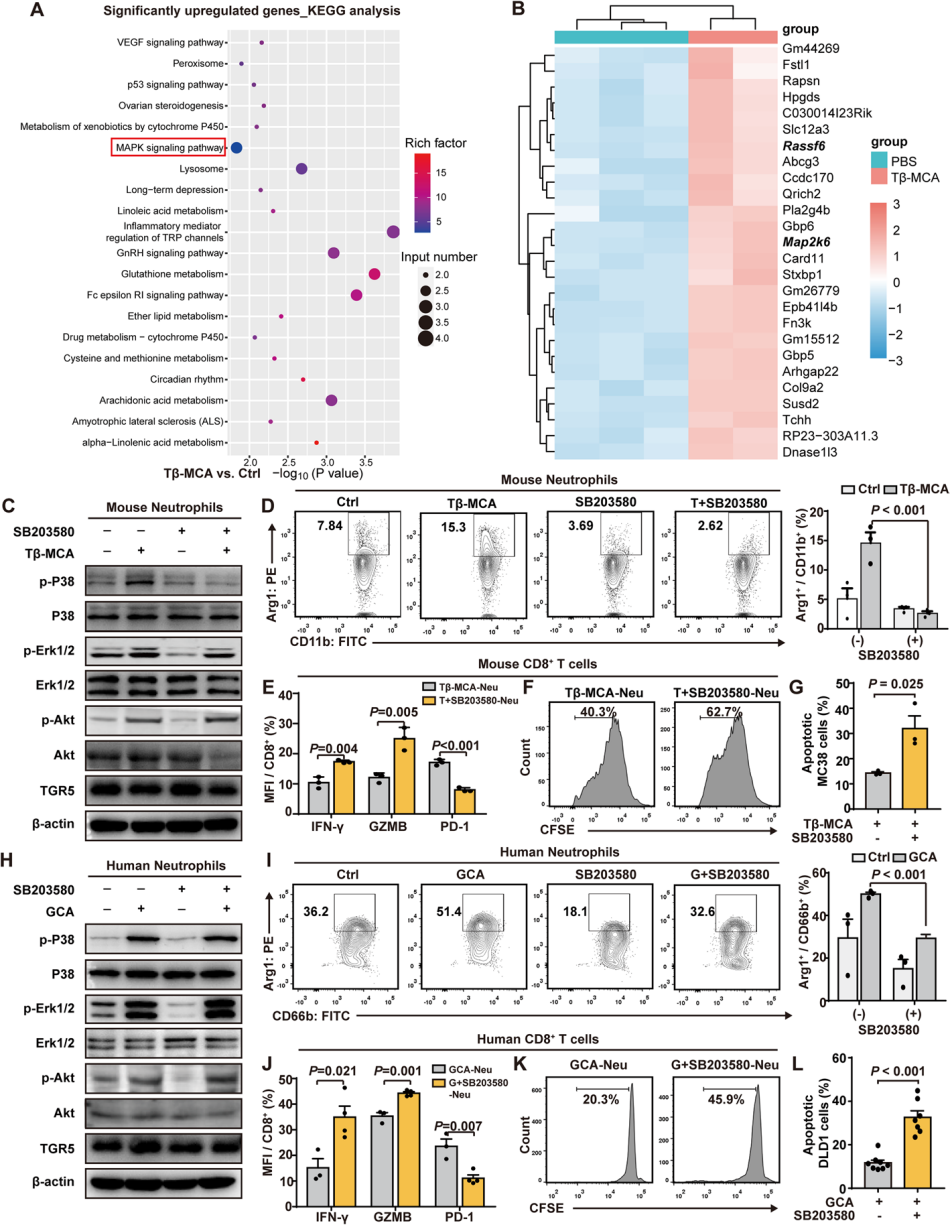
Primary bile acids mediate the immunosuppressive phenotypic-transition of neutrophils via activating the p38 MAPK signaling pathway. **A**-**B** Mouse neutrophils treated with Tβ-MCA were performed with RNA-Seq. KEGG pathway analysis (**A**) and heatmap (**B**) of differentially expressed genes are shown. **C** Western blot analysis of the expression of TGR5, p38, p-p38, Erk1/2, p-Erk1/2, Akt and p-Akt of mouse neutrophils stimulated with Tβ-MCA, SB203580, and both. **D** Flow cytometric analysis of Arg1 expression of mouse neutrophils treated as indicated (*n* = 3 per group). **E** Quantitation of IFN-γ, GZMB, and PD-1 expression of mouse CD8^+^ T cells after coculturing with Tβ-MCA-pretreated neutrophils for 24 h (*n* = 3 per group). **F** Flow cytometric analysis of the proliferation of mouse CD8^+^ T cells after coculturing with Tβ-MCA-pretreated neutrophils alone (left) and with the addition of SB203580 (right) for 24 h (*n* = 3 per group). **G** Flow cytometric analysis of apoptosis of MC38 cells induced by CD8^+^ T cells co-cultured with neutrophils pretreated with Tβ-MCA with or without the addition of SB203580 (*n* = 3 per group). **H** Western blot analysis of the expression of TGR5 and phosphorylated p38 (p-p38), Erk1/2 (p-Erk1/2), and Akt (p-Akt) of human neutrophils stimulated with GCA, SB203580, and both. I, Flow cytometric analysis of Arg1 expression of human neutrophils stimulated with GCA, SB203580, and both (*n* = 3 per group). **J** Quantitation of IFN-γ, GZMB and PD-1 expression of human CD8^+^ T cells after coculturing with GCA-pretreated neutrophils for 24 h (*n* = 3 per group). **K** Flow cytometric analysis of the proliferation of human CD8^+^ T cells after coculturing with GCA-pretreated neutrophils alone (left) and with the addition of SB203580 (right) for 24 h (*n* = 3 per group). **L** Flow cytometric analysis of apoptosis of DLD1 cells induced by human CD8^+^ T cells co-cultured with human neutrophils pretreated with GCA with or without the addition of SB203580 (*n* = 8 per group). Data represent mean ± SEM of 3 biologically independent experiments (Two-tailed t test)

Next, we investigated the mechanism of the phenotypic transition of neutrophils induced by BAs. By analyzing the RNA-seq results of neutrophils treated with TCA or Tβ-MCA, we discovered that MAPK signaling pathway was significantly activated in the Tβ-MCA-treated neutrophils (Fig. 4A, B). MAPK signaling is vital for the regulation of the formation of cathepsin C-mediated neutrophil extracellular trap (CTSC-NET) and the production of reactive oxygen species (ROS) in neutrophils that promotes lung metastases [49]. Upon Tβ-MCA stimulation, p38 and ERK phosphorylation in neutrophils was significantly increased, which could be partially reversed by the p38 inhibitors SB203580 and SB202190 (Fig. 4C, Supplementary Fig. S10A). No significant change was observed in the AKT phosphorylation or TGR5 expression (Fig. 4C, Supplementary Fig. S10A). Furthermore, Tβ-MCA and GCA stimulation on neutrophils increased their Arg1 expression, which was also abrogated by SB203580 and SB202190 treatment (Fig. 4D, Supplementary Fig. S10B-10C). On the other hand, IFN-γ and GZMB derived from CD8^+^ T cells were suppressed while PD-1 expression increased after co-culturing with neutrophils that are pre-stimulated with Tβ-MCA (Fig. 4E, Supplementary Fig. S10D–10E). Meanwhile, the proliferation and cytotoxic effect of CD8^+^ T cells were also inhibited (Fig. 4F, G, Supplementary Fig. S10F-10H). Applying p38 inhibitors restored the cytotoxic effect of CD8^+^ T cells (Fig. 4G, Supplementary Fig. S10H). Similar results were obtained in human neutrophils and CD8^+^ T cells following GCA and p38 inhibitor treatment (Fig. 4H–L, Supplementary S10I–10P). These results implied that the BA-mediated immunosuppressive phenotypic alteration of neutrophils occurred via the p38 MAPK signaling.

Together, these results indicate that primary BAs in cholestasis regulate the recruitment and phenotypic transition of neutrophils, which negatively mediate the activation of lymphocyte and anti-tumor immune responses, thereby creating an immunosuppressive microenvironment that eventually leads to the advancement of LM in CRC.

### OCA treatment reduces the CRC LM associated with cholestasis and enhances the efficacy of the immune checkpoint blockade

Since increased infiltration of neutrophils is caused by cholestasis-induced inflammatory liver injury, we wonder whether controlling the accumulation of bile acids during cholestasis could improve outcomes of MC38-bearing BDL mice. To alleviate cholestasis, Obeticholic acid (OCA) was administrated to in the BDL-subjected mice (Supplementary Fig. S11A). OCA, a synthetic farnesoid X receptor (FXR) agonist that inhibits the synthesis of endogenous BAs, has been approved by the FDA for the treatment of primary biliary cholangitis [50]. OCA successfully decreased pro-inflammatory cytokine production and preserved epithelial barrier function in DSS- and 2,4,6-trinitrobenzene sulfonic acid (TNBS)-induced colitis mouse models [51]. As expected, the plasma level of AST, ALT, TBIL, and DBIL were reduced in OCA-treated BDL mice (Supplementary Fig. S11B). OCA treatment also reduced the development of LM in MC38-bearing BDL mice (Supplementary Fig.S11C)

We further investigated whether targeting BA synthesis restores the activated immune microenvironment. Under OCA treatment alone, the proportion of total CD8^+^ and CD4^+^ T cells, instead of effector memory CD4^+^ T cells, were remarkably elevated in both the liver and peripheral blood (Supplementary Fig. S11D-11F). Furthermore, the proportion of PD1^+^CD8^+^ T cells in cholestatic LM was also reduced under OCA treatment (Supplementary Fig. S11G), indicating the exhaustion of CD8^+^T cells of BDL mice were attenuated via the suppression of bile acid accumulation. The infiltration of neutrophils (Supplementary Fig. S11H) and mRNA levels of Arg1 and iNOS of neutrophils (Supplementary Fig.S11I) were significantly reduced by OCA treatment. Therefore, these findings confirmed that alleviation of cholestasis can reverse the immune-suppressive phenotypic alteration of neutrophil, which in turn recover the immune microenvironment of the liver and abrogate CRC LM development and prolong survival.

**Fig. 5:**
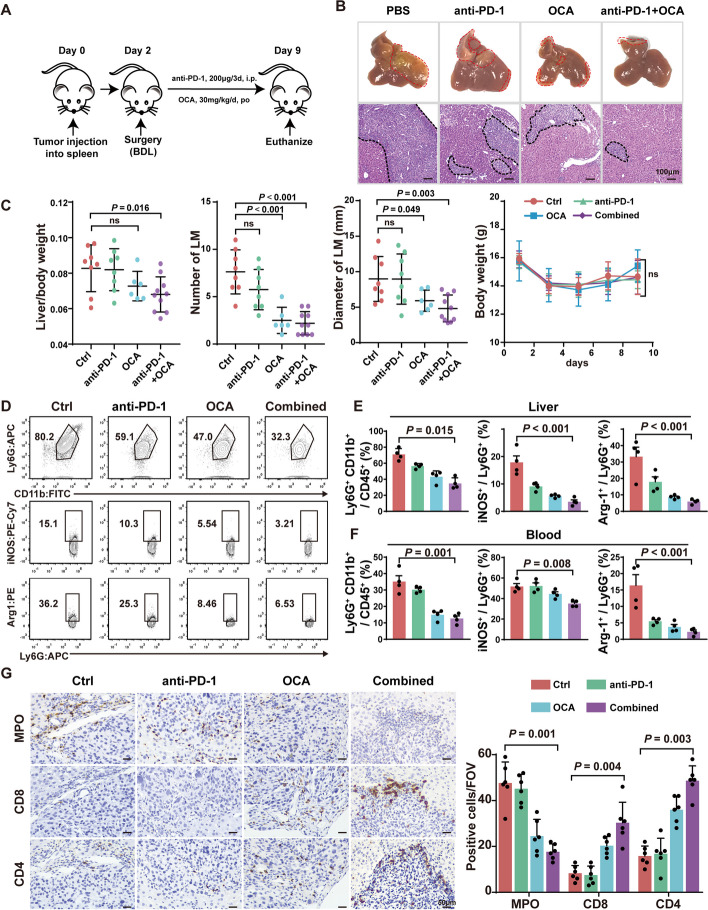
OCA treatment reverses the progression of cholestasis-associated liver metastasis and enhances efficacy of anti-PD-1 immunotherapy. **A** C57BL/6 mice received BDL surgery and liver capsule injection of MC38 cells simultaneously on day 0. Then, the mice received PBS 100 mL, OCA (30 mg/kg, po, qd), anti-PD-1 (200μg/3d, i.p.), and combined therapy before being sacrificing on day 9. **B** Representative images and HE-staining of LM from mice receiving treatments as described. **C** Quantitation of liver/body weight ratio, diameters of LM, tumor nodules and body weight of MC38-bearing BDL mice receiving treatment as described (*n* = 4 per group). **D**-**F** Flow cytometry analysis (**D**) and quantification (**E**, **F**) of the expression of Arg1, iNOS of neutrophils in liver and blood of mice receiving treatments as described. **G** Representative images and quantification of immunohistochemical staining of MPO, CD4 and CD8 of LM of mice under treatments as described (*n* = 6 per group). Data represent mean ± SEM of 3 biologically independent experiments (Two-tailed t test and one-way ANOVA). ns, no significance. Ctrl, control; OCA, obeticholic acid; anti-PD-1, anti-PD-1 antibody; Combined, OCA feeding plus anti-PD-1 treatment

T cell exclusion within the liver metastatic niche often leads to poor immunotherapeutic effects [52]. However, it remains unclear whether cholestasis influences the susceptibility of colorectal LM to immunotherapy. To study whether the alleviation of cholestasis can improve the therapeutical outcome of immune checkpoint blockade, we applied OCA treatment alone, anti-PD-1 treatment alone, and combination of OCA and anti-PD-1 to MC38-bearing BDL mice (Fig. 5A). As expected, the combination of OCA and anti-PD-1 was the most effective regimen in inhibiting tumor growth, whereas anti-PD-1 alone did not (Fig. 5B, C). In addition, either single or combined treatment with OCA resulted in little change in the body weight of mice, suggesting the safety and clinical implications of these therapeutic regimens. Moreover, combined treatment of OCA and anti-PD-1 achieved the greatest effect of suppressing the accumulation of neutrophils and their expression of Arg1 and iNOS both in liver and blood (Fig. 5D-F), while promoting infiltration of CD8^+^ and CD4^+^ T cells, which was an outcome that the treatment of OCA or anti-PD-1 monotherapy could not achieve (Fig. 5G).

These results suggest that OCA treatment could potentiate the efficacy of ICB by promoting the infiltration and transition of T cells and neutrophils, providing novel strategies for the treatment of CRC LM associated with cholestasis.

### Cholestasis is associated with an immunosuppressive microenvironment and poor therapeutic efficacy in CRC patients with LM

**Fig. 6:**
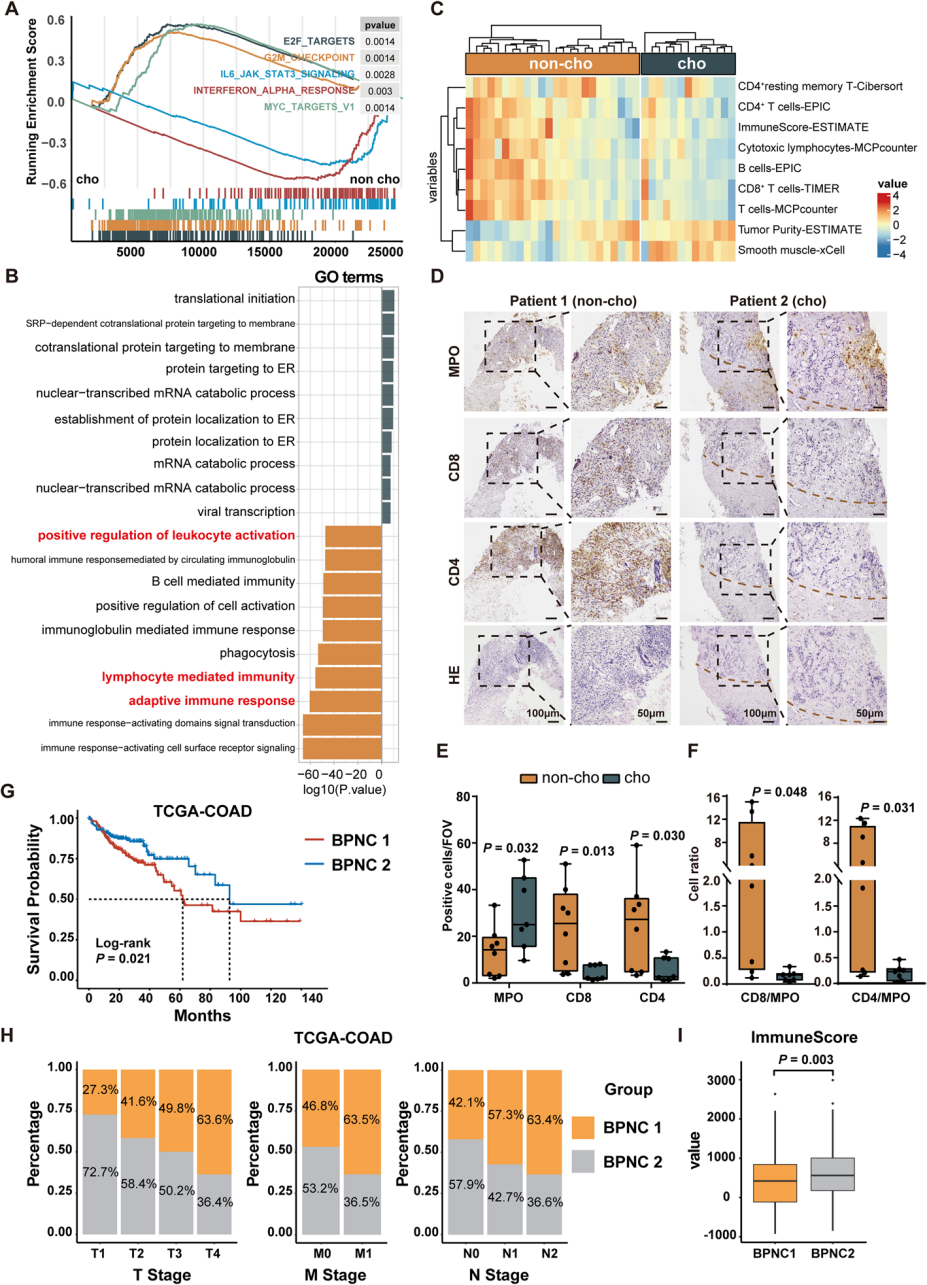
Clinical data confirm the immunosuppressive microenvironment of cholestatic liver metastasis. Samples of non-cholestatic (*n* = 24) and cholestatic (*n* = 13) colon cancer LM were collected for RNA-seq. **A** GSEA enrichment plots showing the IFN-α response, IL6/Stat3 signaling, E2F targets, G2M checkpoint, and Myc target gene sets in non-cholestatic and cholestatic patients with LM. **B** GO analysis of differentially expressed genes in non-cholestatic and cholestatic patients with LM. **C** Heatmap of TME cells in non-cholestatic and cholestatic patients with LM were generated using various immune cell evaluation tools. **D**-**F** Representative images (**D**) and quantitation (**E**, **F**) of immunohistochemical staining of MPO, CD4 and CD8 of LM of non-cholestatic (*n* = 8) and cholestatic (*n* = 7) patients. Data represent mean ± SEM of 3 biologically independent experiments (Two-tailed t test). **G** Kaplan–Meier analysis of the overall survival of TCGA-COAD patients classified as “BPNC 1” and “BPNC 2” group. Log-rank test. **H** TNM percentage analysis of “BPNC 1” and “BPNC 2” group patients in TCGA-COAD. **I** ImmuneScore analysis of “BPNC 1” and “BPNC 2” group patients in TCGA-COAD. Cho, cholestasis; non-cho, non-cholestasis. BPNC, BA-P38-Neu cluster

To further confirm the in vitro and in vivo results in patients, we performed RNA sequencing analysis of puncture biopsy specimens from patients with CRLM (*n* = 13 for cholestasis; *n* = 24 for non-cholestasis). Consistent with previous results, GSEA and GO analysis demonstrated that the immune activation-related pathways of leukocyte activation, lymphocyte activation, adaptive immune response, and interferon-alpha response were significantly downregulated in the cholestasis group as compared to those in the non-cholestasis group (Fig. 6A-B). Furthermore, patients with cholestatic LM exhibited decreased CD8^+^ and CD4^+^ T cell infiltration and a low immune score, which was analyzed by EPIC, TIMER, and CIBERSORT algorithms (Fig. 6C), which was also validated by immunohistochemistry (Fig. 6D-F) performed on puncture biopsy specimens from patients.

Given the above experimental findings, we aim to demonstrate the significance of the bile acid excessive-characteristic liver niche in cancer patients. To establish a specific gene signature to classify CRC patients, gene signatures of bile acid metabolism of MSigdb database, p38 MAPK signaling pathway of Genecard, and gene signatures of tumor-associated neutrophils of Zhang etl [23] were collected. The selected genes were performed with univariate Cox analysis and intersected with DEGs of The TCGA human colon cancer dataset (TCGA-COAD) to filter out those that significantly correlated with survival of TCGA-COAD, which generated a final gene list of 8 genes, termed the “BA-P38-Neu gene set”. Unsupervised clustering (K means) based on the expression of the eight genes classified the patients in TCGA-COAD into two distinct clusters, termed as “BA-P38-Neu cluster 1 (BPNC1)” and “BA-P38-Neu cluster 2 (BPNC2)” group. As is shown, the overall survival (OS) of the “BPNC1” group was significantly shorter than that of the “BPNC2” group (Fig. 6G). Furthermore, “BPNC 1” group was correlated with a higher TMN stage of colon cancer (Fig. 6H). Furthermore, the ImmuneScore [25] of the “BPNC 1” group is significantly lower than the “BPNC 2” group patients, indicating the distinctive immune microenvironment of the two groups (Fig. 6I). Accordingly, we concluded that the integrated gene set related to these three signaling pathway mentioned above could effectively identify patients with potential of better clinical outcomes, which will further contribute to more precise treatment strategies for colorectal cancer patients.

Collectively, these clinical data further support that cholestasis-induced neutrophil’s phenotypic transformation contributes to the development of the ‘immune-desert’ environment and progression of tumors.

## Discussion

Cholestasis, resulting from intrahepatic or extrahepatic bile obstruction, is a major complication of LM development. CRLM patients with cholestasis could not receive further administration of systemic therapy until normal liver function is restored. Even though some patients with malignant biliary obstruction can benefit from biliary drainage, their overall survival and life quality is remarkedly declined. Effective therapeutic strategies such patients are still lacking [3]. Although increasing evidence has shown that cholestasis is associated with immunological abnormalities, such as metabolic and autoimmune diseases, the impact of cholestasis on cancer and the TME remains unclear [53–55]. Here, we show that primary BAs accumulated during cholestasis contributed to the development of LM by recruiting neutrophils and promoting their immunosuppressive phenotype, resulting in impaired adaptive immune response and an immunosuppressive microenvironment.

In the present study, we have demonstrated that, regardless of the onset time or precise site of biliary obstruction, cholestasis contributes to the progression of LM of CRC and worsen survival, by investigating various tumor-bearing cholestatic mouse models and clinical data. Although sample size was small, our clinical data showed a significant reduction in the progression-free survival of CRC patients with LM and cholestasis. The RNA-Seq analysis from both *in vivo* assays and clinical samples surprisingly found that the DEGs were not enriched in tumor proliferation or metastasis pathways, but were enriched in tumor immunity and microenvironment-related signaling. Animal studies confirmed that the activation and infiltration of T cells were also inhibited in the CRC LM associated with cholestasis. These results suggested that an abnormal immune microenvironment, instead of endogenous changes in the tumor cells, was critical for the induction of cholestasis associated LM in CRC.

In the present study, we discovered that the recruitment of neutrophils and the exclusion and inactivation of lymphocytes were the main characteristics of the CRC LM immune microenvironment associated with cholestasis. Tumor-associated neutrophils (TANs), which are important components of the TME, can affect the progression of LM in all solid tumors in various ways, including malignant transformation, tumor progression, ECM modification, angiogenesis, cell migration, and immunosuppression. Generally, TANs can be divided into mature and immature types, of which immature TANs show a preference for immunosuppressive phenotypes and contribute to tumor development [56–58]. Interestingly, in the present study, neutrophils infiltrating in the CRC LM associated with cholestasis showed decreased leukocyte differentiation and activation with increased expression of *Arg1* and *NOS2*. In vivo experiments showed that BAs-stimulated neutrophils suppressed the activation of T cells. indicating an immunosuppressive phenotype of such TANs. As a result, it is reasonable to speculate that immune-suppressive neutrophils were the main cause of cholestasis-induced suppression of the TME and LM progression.

Moreover, despite effectively inhibiting the CRC LM by restoring the immune-hot microenvironment, neutrophil depletion, unfortunately, accelerated animal death and failed to achieve clinical applicability, which may be attributed to the deficient innate immune defense provided by neutrophils during cholestasis that eventually lead to systemic inflammation and infection [59, 60]. Consequently, we focused on the transition, and not the elimination of neutrophils in the present study.

Previous studies on the phenotypic transition of neutrophils have mainly focused on cytokines [61]. However, BA metabolism disorders are the main cause of cholestasis resulting in a significant increase in the levels of primary BAs in the liver [46]. BAs not only play an important role in lipid metabolism, but also act as signaling molecules to regulate energy metabolism, immunity, and inflammation by activating the BA receptor (GPBAR-1, also known as TGR5) and Farnesis-X-receptor α (NR1H4) [62, 63]. The taurodeoxycholate (TDCA) can significantly activate TGR5-cAMP signaling in myeloid inhibitory cells (MDSCs) to inhibit inflammatory responses and prolong survival during sepsis [64]. Our results also confirmed our hypothesis that Tβ-MCA and GCA could effectively promote the phenotypic transformation of neutrophils through the p38 MAPK signaling axis, thus, affecting the activation and cytotoxic effects of T lymphocytes. Furthermore, the inhibition of BA synthesis and secretion by OCA can reverse the immunosuppressive microenvironment of LM and increase the efficacy of immunotherapy in cholestasis presenting CRC patients. Examination of patients’ tissue tumor sections also verified the infiltration of neutrophil in cholestatic patients. Moreover, the integrated gene set of “BA-P38-Neu” can classify colon cancer patients into distinctive survival groups, indicated its translational potential and clinical significance for therapy. To indicate the activation of p38 MAPK signaling pathway, simply investigating the p38 phosphorylation in tumor of patients to predict survival of colorectal cancer patients is not applicable in clinical practice, since the p38 phosphorylation is a kind of protein modification of p38 MAPK, which is not as convenient as investigating mRNA expression. On the other hand, the "BA-P38-Neu gene set" can serve better in classifying patients and predicting the survival of colon cancer patients, because it is a comprehensive and integrated gene set that not only contains the p38 gene, but also genes involved in the p38 MAPK signaling pathway cascade as well as correlating with the signatures of neutrophils and bile acid metabolism.

Our results indicate that cholestasis and LM intricately interact and influence each other, exhibiting a cause-and-effect relationship. The clinical applications of OCA should be comprehensively explored in future studies to ascertain its clinical efficacy in the treatment of CRC patients with LM and cholestasis.

## Conclusion

This study revealed that cholestasis accelerated the progression of colorectal cancer liver metastasis. By focusing on the TME change, we demonstrated that liver injury, neutrophil infiltration and T cell exclusion and dysfunction contributed to the development of liver metastasis. Mechanistically, primary bile acid TβMCA and GCA drived the immunosuppressive phenotypic transformation of neutrophil via activating p38 MAPK signaling. Reversing the excessive accumulation of bile acids by Obeticholic acid could suppress tumor growth and improve the effect of immunotherapy. This study proposed a new strategy for such patients by targeting bile acid anabolism.

### Supplementary Information

Supplementary Material 1. 

Supplementary Material 2. 

Supplementary Material 3. 

Supplementary Material 4. 

Supplementary Material 5. 

Supplementary Material 6. 

Supplementary Material 7. 

Supplementary Material 8. 

Supplementary Material 9. 

Supplementary Material 10. 

Supplementary Material 11. 

Supplementary Material 12. 

## Data Availability

The RNA-Seq data of neutrophils stimulated by Tβ-MCA and TCA is available in the GEO database (accession number GSE209836). The RNA-Seq data of LM of sham and BDL-subjected mice or samples from colorectal cancer patients are not available due to conduct of further studies.

## Abbreviations

ANIT: α-naphthyl isothiocyanate
BAs: Bile acids
BDL: Bile duct ligation
CRC: Colorectal cancer
DBIL: Direct bilirubin
GO: Gene ontology
GSEA: Gene set enrichment analysis
ICB: Immune checkpoint blockade
LM: Liver metastasis
mCRC: Metastatic colorectal cancer
OCA: Obeticholic acid
PCA: Principal component analysis
TBIL: Total bilirubin
TME: Tumor microenvironment
