# Supplementary figures and images for: Cholestasis-induced phenotypic transformation of neutrophils contributes to immune escape of colorectal cancer liver metastasis
Source: J Biomed Sci. 2024 Jun 29;31:66. doi: 10.1186/s12929-024-01052-3 (PMC11218316; doi:10.1186/s12929-024-01052-3)

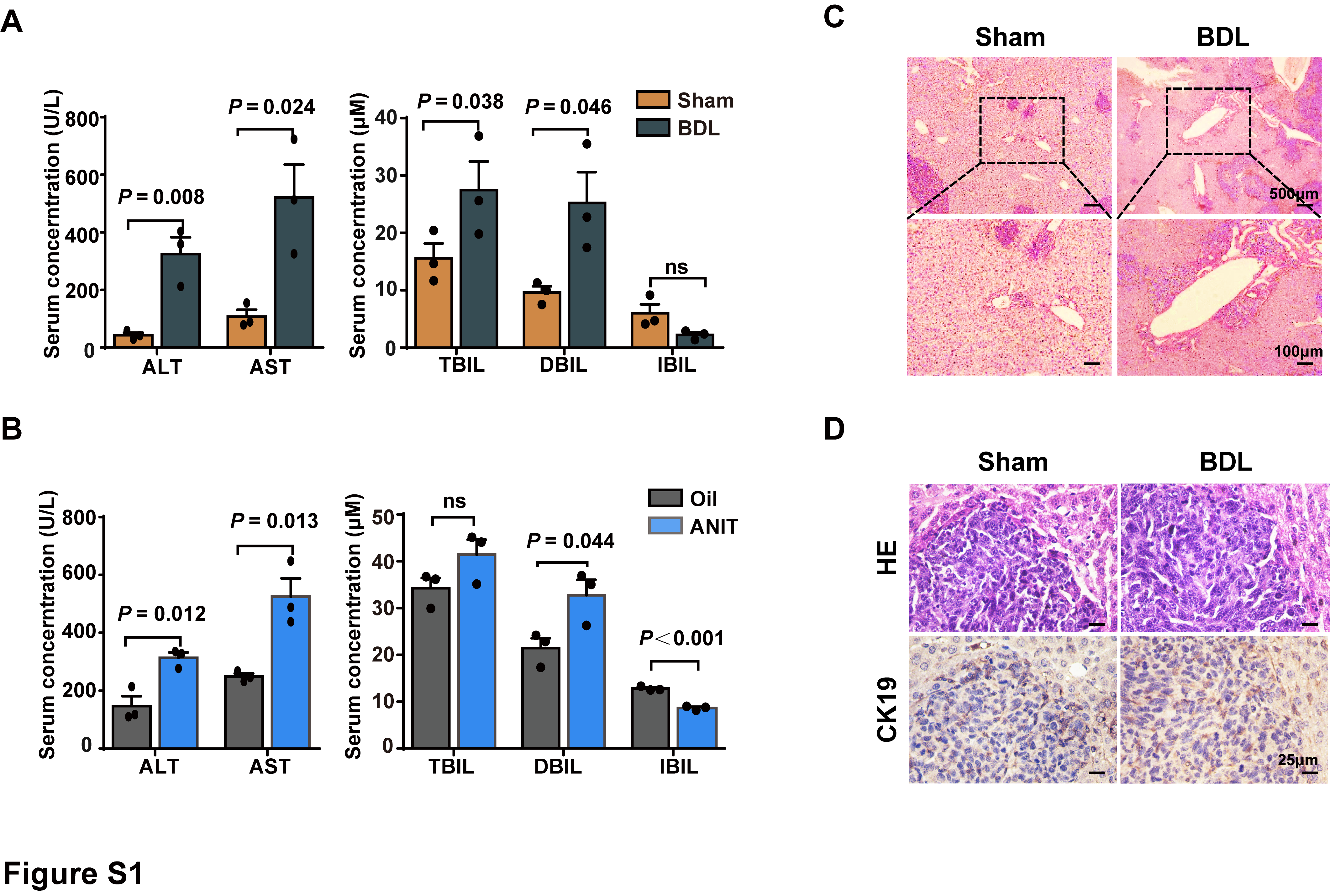

Supplement: Supplementary file 1 — Supplementary Material 1. [file 12929_2024_1052_MOESM1_ESM.tif]

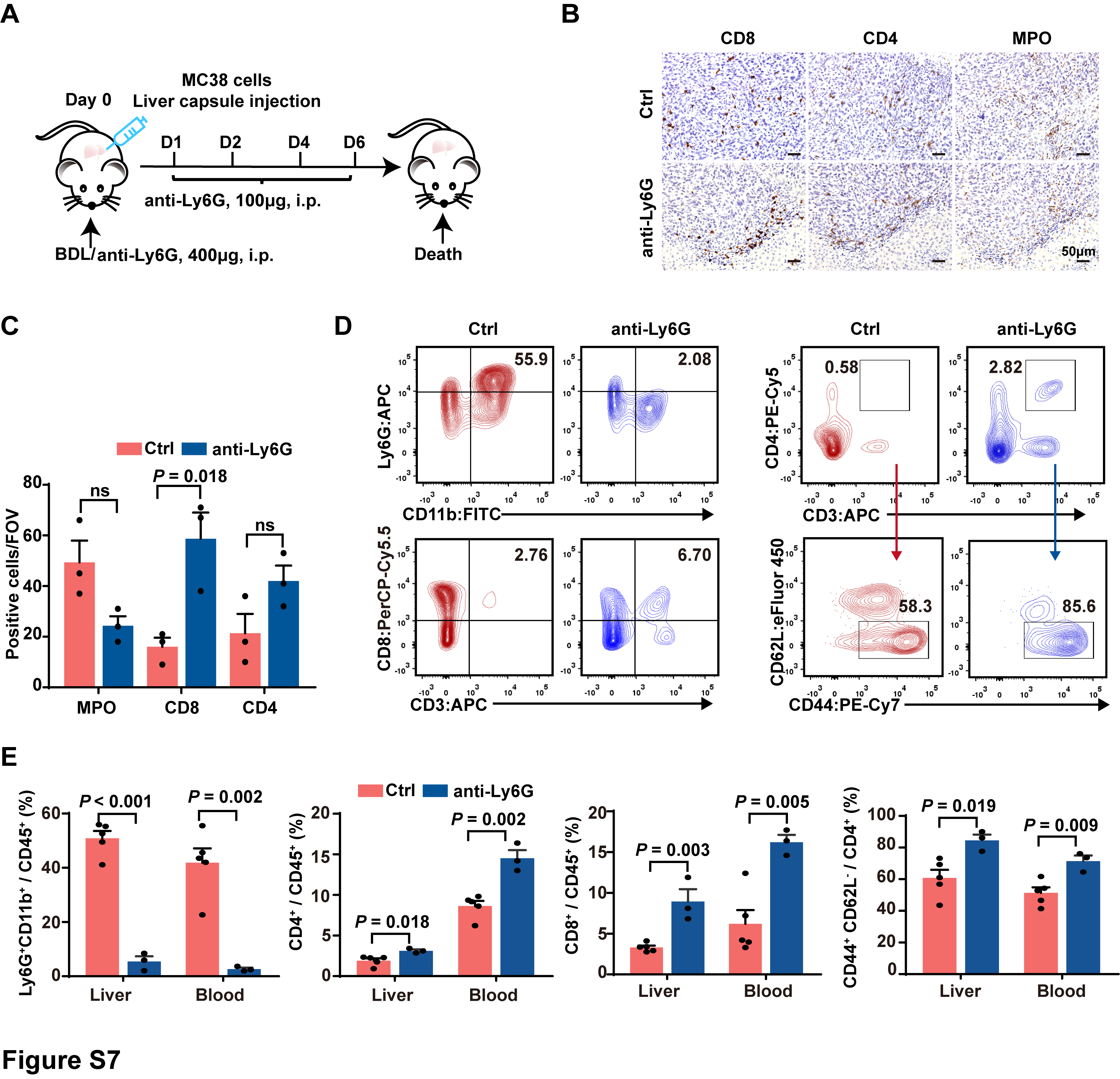

Supplement: Supplementary file 7 — Supplementary Material 7. [file 12929_2024_1052_MOESM7_ESM.tif]
