# Supplementary material for: Cholestasis-induced phenotypic transformation of neutrophils contributes to immune escape of colorectal cancer liver metastasis
Source: J Biomed Sci. 2024 Jun 29;31:66. doi: 10.1186/s12929-024-01052-3 (PMC11218316; doi:10.1186/s12929-024-01052-3)

Supplementary

Original blot

Fig 4E

Tβ-MCA SB203580

Lane 1 p-p38





Lane 2 p38





Lane 3 p-ERK1/2


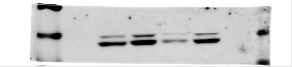


Lane 4 ERK1/2





Lane 5 p-Akt


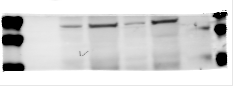


Lane 6 Akt





Lane 7 TGR5


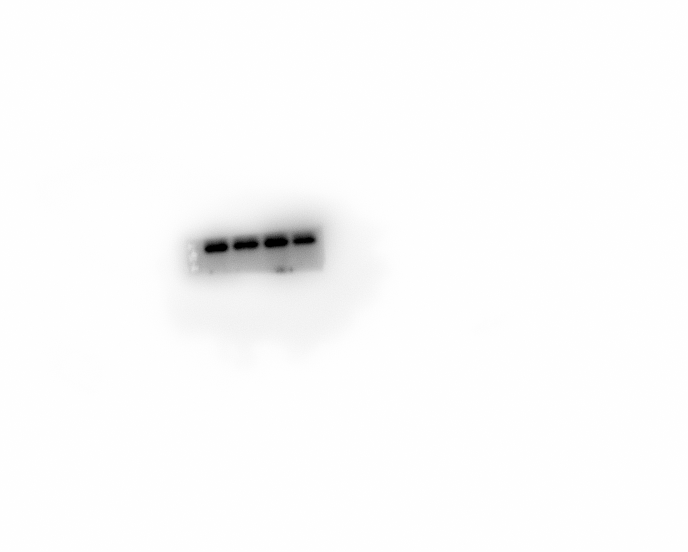


Lane 8 actin


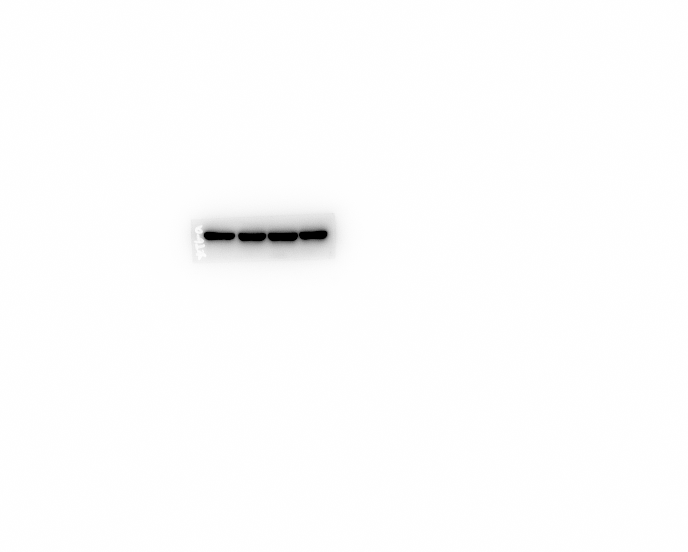


Figure 4H

GCA SB203580

Lane 1 p-p38





Lane 2 p38





Lane 3 p-ERK1/2





Lane 4 ERK1/2





Lane 5 p-Akt


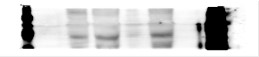


Lane 6 Akt


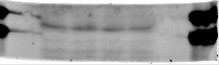


Lane 7 TGR5





Lane 8 actin





Supplementary Figure S8A

Tβ-MCA SB202190

Lane 1 p-p38


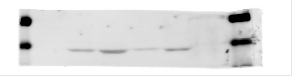


Lane 2 p38


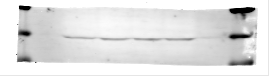


Lane 3 p-ERK1/21/2


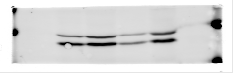


Lane 4 ERK1/21/2


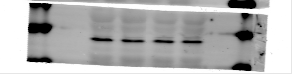


Lane 5 p-Akt


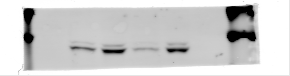


Lane 6 Akt


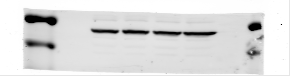


Lane 7 TGR5


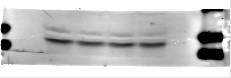


Lane 8 actin


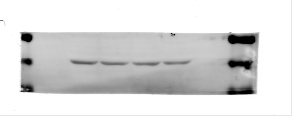


Supplementary Figure S8I

GCA SB202190

Lane 1 p-p38


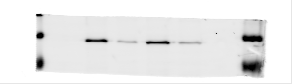


Lane 2 p38


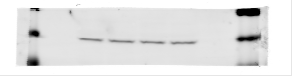


Lane 3 p-ERK1/21/2


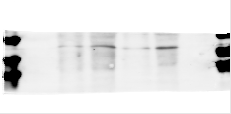


Lane 4 ERK1/21/2


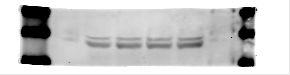


Lane 5 p-Akt


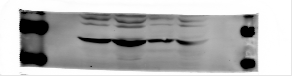


Lane 6 Akt


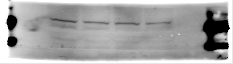


Lane 7 TGR5


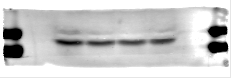


Lane 8 actin


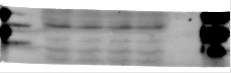

Supplement: Supplementary file 12 — Supplementary Material 12. [file 12929_2024_1052_MOESM12_ESM.docx]
